# A new brace treatment similar for adolescent scoliosis and kyphosis based on restoration of thoracolumbar lordosis. Radiological and subjective clinical results after at least one year of treatment

**DOI:** 10.1186/1748-7161-7-19

**Published:** 2012-10-29

**Authors:** Piet JM van Loon, Monique Roukens, Joop DJ Kuit, Frederik BTM Thunnissen

**Affiliations:** 1 Department Orthopaedic Surgery, Gelre Ziekenhuizen Apeldoorn, Albert Schweitzerlaan 31, 7300, DS, Apeldoorn, The Netherlands; 2 Dept.Universitair Medical Centre, Radboud University Nijmegen, Geert Grooteplein zuid 10, 6525, GA, Nijmegen, The Netherlands; 3 Department of Medical Epidemiology, Free University Amsterdam, De Boelelaan 1117, 1081, HV, Amsterdam, The Netherlands; 4 Department of Pathology, Free University Amsterdam, De Boelelaan 1117, 1081, HV, Amsterdam, The Netherlands

## Abstract

**Study design:**

A prospective treatment study with a new brace was conducted Objective. To evaluate radiological and subjective clinical results after one year conservative brace treatment with pressure onto lordosis at the thoracolumbar joint in children with scoliosis and kyphosis.

**Summary of background data:**

Conservative brace treatment of adolescent scoliosis is not proven to be effective in terms of lasting correction. Conservative treatment in kyphotic deformities may lead to satisfactory correction. None of the brace or casting techniques is based on sagittal forces only applied at the thoracolumbar spine (TLI= thoracolumbar lordotic intervention). Previously we showed in patients with scoliosis after forced lordosis at the thoracolumbar spine a radiological instantaneous reduction in both coronal curves of double major scoliosis.

**Methods:**

A consecutive series of 91 children with adolescent scoliosis and kyphosis were treated with a modified symmetric 30 degrees Boston brace to ensure only forced lordosis at the thoracolumbar spine. Scoliosis was defined with a Cobb angle of at least one of the curves [greater than or equal to] 25 degrees and kyphosis with or without a curve <25 degrees in the coronal plane. Standing radiographs were made i) at start, ii) in brace at beginning and iii) after one year treatment without brace.

**Results:**

Before treatment start ‘in brace’ radiographs showed a strong reduction of the Cobb angles in different curves in kyphosis and scoliosis groups (sagittal n = 5 all p < 0.001, pelvic obliquity p < 0.001). After one year of brace treatment in scoliosis and kyphosis group the measurements on radiographs made without brace revealed an improvement in 3 Cobb angles each.

**Conclusion:**

Conservative treatment using thoracolumbar lordotic intervention in scoliotic and kyphotic deformities in adolescence demonstrates a marked improvement after one year also in clinical and postural criteria. An effect not obtained with current brace techniques.

## Background

The conservative treatment with braces of adolescent spinal deformities in modern times, especially scoliosis, has not proven to be effective in terms of lasting correction of curves. In scoliosis several studies can show positive results in preventing progression of curves [1-4]. In comparison with the ample literature for bracing in scoliosis, for kyphotic deformities only scarce literature on conservative treatment is available. But in contrast to scoliosis, in kyphotic deformities real correction of curves with casting and bracing has been shown [5-10]. In the SOSORT 2005 Consensus Paper: “Brace action: where to push and why” the members give an oversight of all used types of rigid braces used in scoliosis in their institutions and the controversial factors in their way of acting and effectiveness, but all used pressure from lateral at the height of the apex [11]. All types of rigid bracing techniques were recently reviewed [12,13]. No brace technique described in present literature is based on pure sagittal forces applied only at the thoracolumbar spine, thereby extending the spine as a whole. There are no studies available on a bracing technique equal for both of the most prevalent adolescent spinal deformities as the adolescent scoliosis and the adolescent kyphosis or hyperkyphosis, based on the same industrial made module. In a recent radiological study we showed the existence of a kyphosis at the thoracolumbar spine in any double curved scoliosis [14]. In addition, we showed that thoracolumbar lordotic intervention in the sagittal plane with a fulcrum, purely at the thoracolumbar joint, resulted in reduction of the deformities in the other planes and other areas in the sagittal contour. We concluded that pressure onto lordosis at the thoracolumbar joint seems more effective than our earlier used three point correction with pressure at the apex of the scoliotic curves. In this published study the immediate reduction also occurred in serious curves and almost matured children. The notion that restoration of lordosis at the thoracolumbar joint may be beneficial in deforming spines raised the question whether prolonged lordotic support by a thoracolumbar lordotic intervention in bracing may be beneficial throughout the process of growth. A brace which give children an immediate feeling of a correct posture seemed to be the answer. Because the restoration towards a normal or good posture in morphologic and functional ways was part of this technique, efforts were made to look for better etiologic factors or original science on this, that seems to be addressed more properly by this technique. The literature search for scientific background of deformities and its support to the proposed way of action to correct these disturbances of growth is described separately. The aim of this pilot study was to evaluate conservative treatment with this bracing technique in children after one year of treatment in the normal way by assessing radiographs. To this end the effect of a thoracolumbar lordotic intervention (TLI) in scoliotic and kyphotic deformities in adolescence was radiological measured. In addition, after one year patient satisfaction was recorded. Most of the radiological curves in coronal and sagittal planes improved strongly at the beginning and were still significant after one year of treatment.

The amount of children that completed brace treatment at time of this study and were seen 1 year after stopping was with 5 to small to be included. The reason only results after one year or preliminary results could be presented is a local one: the main author changed for institution and encountered serious inhibititive forces to proceed with this research. All children completed the regimen, but the results after one year provide good statistical insight in the potentials of this technique of treatment. An attempt to undertake a long term follow up of this cohort in that former institution, that skipped all care for spinal deformities is under preparation.

## Materials and methods

### Patients

In our (former) institution a consecutive series of 91 children with adolescent spinal deformities, scoliosis and kyphosis were prospectively treated with a thoracolumbar lordotic intervention brace for at least one year in the period 2002–2005. Two groups were formed (Table 1): Group A with a predominantly scoliotic deformity in which the Cobb angle of at least one of the curves was more or equal than 25° and Group B with predominantly kyphotic deformity without scoliosis or scoliotic curves of less than 25°. 8 children in group A did wear a conventional Boston brace prescribed in other institutions and were referred because of progression.

**Table 1:** Clinical characteristics and distribution into group A and B based on coronal Cobb angle is shown

**Characteristics of: deformity patients**	**Group A scoliosis (>25°)**		**Group B kyphosis (scoliosis <25°)**		**Total**	
	**n**	**%**	**n**	**%**	**n**	**%**
**Gender**						
Boy	8	21.1	44	55.7	45	49.5
Girl	30	78.9	35	44.3	46	50.5
**AGE at start of brace**	13.5±2.0		13.9±1.9			
**Menarche**	12.7±1.2		12.9±1.1			
**Presence of Scoliosis (≥ 25°)**						
Yes	38	100.0	26	32.9	38	41.8
No	0	0.0	53	67.1	53	58.2
**Presence of Kyphosis**						
Yes	26	31.6	79	100.0	79	86.8
No	12	68.4	0	0.0	12	13.2

n = number of patients; age: mean ± standard deviation.

Both growth dependant deformities were put together for several reasons, including the fact they react from the start (partly found by serendipity) the same on this type of correction and share most of their etiologic factors in the neuro-musculoskeletal integrity of growth processes and are based on the same early kyphotic changes in children with sitting as the prevailing posture in their lives.

Inclusion criteria:

Structural scoliosis or Thoracic hyperkyphosis with/or the presence of a Structural thoracolumbar kyphosis. The magnitude of the coronal nor the sagittal curves was not limited to a maximum in this study.

Exclusion criteria:

All magnitudes of maturation were allowed, except Risser V. Evident neuromuscular deformities, as in Cerebral Palsy and Meningomyelocele, congenital Deformities or concomitant syndromes as Marfan or Prader Willie.

### The brace technique

**Figure 1:**
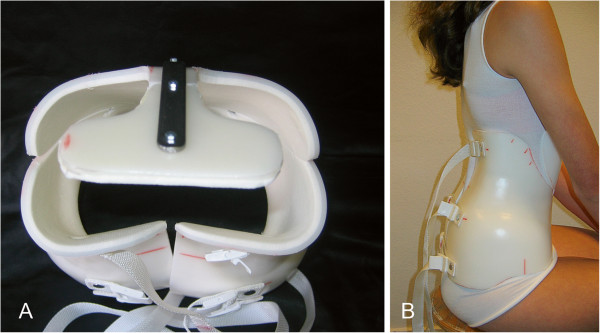
**An example of a TLI brace.** At the left (**A**)a brace seen from above to show the all lordotic configuration. At the back side the curved configuration with the apex at the thoracolumbar area is visible. The pressure points face the erecting muscles and are not directed to bony structures like ribs. At the right (**B**) a 14 year old girl with a double curved scoliosis at time of the first fitting, seen from aside.

For this specific brace technique in all children the 30° lordosis Boston Brace (BBE Healthcare, Dundalk, Ireland) module was ordered and modified by (i) adding a bendable sternal support on the front side, (ii) trimming the body of the brace in a symmetrical way down to the level of the lower ribs, and (iii) reduction on the backside to half of the sacrum, freely supporting the lumbosacral lordotic curvature. No asymmetric pressure on bony structures was used, but (iv) extra padding was placed at the thoracolumbar spine at the paravertebral mass of the musculus erector trunci to create a lordotic fulcrum inside the brace (see Figure 1). This brace treatment is further called thoracolumbar lordotic intervention (TLI). All orthotic work was done by one orthotist (JM†). All children stayed for one night in the hospital in order to get them used to wear the brace under strict conditions.

The recommended brace regimen consisted of wearing the brace at least 18 hours. Besides wearing it at night the child was urged to wear the brace at school to prevent the flexed position of the spine as much as possible. The hours “off-brace” were meant for bathing, sports and the more standing and walking activities. Every 3–4 months patients were controlled and by physician and orthotist for possible modification of the brace in the sense of providing more lordotic pressure. The sternal support was bent backwards each time and at the backside rims were taken of at the upper and the lower side for better extension. Brace treatment was stopped at estimated end of growth: closure of the pelvic apophyses (Risser V).

#### Postural therapy

In all patients postural therapy was applied before and continued during brace treatment. The postural therapy was performed with emphasis on extension and mobilizing the thoracolumbar spine, strengthening of the erecting muscles and stretching of tight musculature (e.g. hamstrings) while preventing flexion in the spine. Advise was given on optimal sitting postures and the need to change postures often. This was consequently prescribed in our institution for more than four decades, also when the previous brace types were in use. In Europe most institutions, certainly those related with SOSORT have exercise therapy incorporated in the conservative treatment in order to reinforce balance, optimal function and condition of the trunk musculature.

#### Radiology

Standing radiographs in AP and lateral direction were made when possible at three time points:

T0 = indication brace treatment ( X-ray without brace, before brace treatment) T1 = start of the brace treatment (“in brace correction”) and T2 = one year after start brace treatment (at least one hour without brace). Cobb method in coronal and sagittal planes on X-rays was used as described previously [14].

#### Questionnaire

After one year a patient questionnaire was taken by an independent researcher. The questions aimed at the perception of treatment effect and information received from different health care workers involved in treatment.

#### Statistical analysis

For group A (N =38 ) and Group B (N = 53) treatment was evaluated by pair wise comparison with students-T test for Cobb angle T0^OB^ with T0^IB^ and at T1. Progression after treatment is defined as an increase 6° in Cobb angle at T1 compared to T0^OB^[15]. From answers in the questionnaire mean and standard deviation were calculated. P<0.05 was considered to be significant.

## Results

A total of 91 patients entered the study: 45 males and 46 females, mean age at start of treatment 13.8 ± 1.9 years and menarche (n = 38) 12.8 ± 1.2 years. With postural assessment at control visits the brace frequently showed some space at the level of the sternal support, indicating increased extension of the spine. In these instances pressure pads at the thoracolumbar area were adjusted (by adding foam liner) in combination with backward bending of the sternal support leading to more spinal lordosis or extension in at least 2 of the 5 visits. The number of scoliosis (group A) and kyphosis (group B) patients was 38 and 53, respectively. 67 out of 91 had evaluable radiological data for at least one Cobb angle. For group A and group B the different angles from ‘in brace’ and treatment comparison are shown in Table 2. The ‘in brace’ results show for 6 out of the 7 angles a significant difference, proving the possibility of improvement towards lasting correction (Wolff’s Law).

**Table 2:** **The mean and standard deviation (mean ± SD) of the difference in Cobb angles is shown separately for the two groups for various curves in two planes for comparison at the start of treatment with and without brace T0**^**OB-IB**^**and after one year of brace treatment and the progression (Prog) rate after one year(T0**^**OB**^**-T1)**

	**Pair**	**Group B kyphosis +/− scoliosis <25°**			**Prog rate**	**Group A scoliosis ≥25°**			**Prog rate**
**Curve localisation**		**n**	**mean ± SD**	**p value**	**n (%)**	**n**	**mean ± SD**	**p value**	**n (%)**
**Thoracic right**	T0 OB-IB	7	1.6 ± 6.6	0.55		29	6.7 ± 1.2	<0.001	
	T0 OB -T1	5	−1.8 ± 3.8	0.35	0 (0%)	28	8.9 ± 1.7	0.38	6 (21%)
**Lumbar left**	T0 OB-IB	4	7.3 ± 6.8	0.12		6	8.0 ± 3.2	0.045	
	T0 OB -T1	3	5.0 ± 2.0	0.049	0 (0%)	6	7.2 ± 2.9	0.53	1 (20%)
**Thoracolumbar left**	T0 OB-IB	9	7.8 ± 6.1	0.005		26	9.0 ± 6.3	<0.001	
	T0 OB -T1	8	1.8 ± 6.7	0.49	1 (12%)	24	0.92 ± 4.8	.36	1 (4.2%)
**Pelvic obliquity**	T0 OB-IB	4	6.5 ± 2.1	0.008		25	3.1 ± 4.1	0.001	
	T0 OB -T1	7	2.8 ± 4.2	0.12	0 (0%)	23	1.1 ± 4.9	0.24	1 (4.3%)
**Thoracic sagittal**	T0 OB-IB	40	13.3 ± 8.9	< 0.001		21	8.2 ± 7.8	<0.001	
	T0 OB -T1	6	15.7 ± 6.2	0.002	0 (0%)	14	9.6 ± 10.0	0.008	0 (0%)
**Thoracolumbar sagittal**	T0 OB-IB	43	7.0 ± 7.1	<0.001		26	6.0 ± 6.7	<0.001	
	T0 OB -T1	10	7.4 ± 6.9	0.008	0 (0%)	20	7.6 ± 6.6	<0.001	0 (0%)
**Lumbar sagittal**	T0 OB-IB	38	8.7 ± 2.7	<0.001		21	6.7 ± 7.7	<0.001	
	T0 OB-T1	6	10.3± 13.6	0.13	0 (0%)	14	2.3 ± 7.1	0.29	3 (21%)
**Pelvic incidence**	T0 OB-IB	36	6.8 ± 6.3	<0.001		21	5.8 ± 6.2	0.001	
	T0 OB -T1	6	9.3 ± 9.8	0.07	0 (0%)	14	5.0 ± 6.3	0.011	0 (0%)
**Sacral inclination**	T0 OB-IB	36	2.6 ± 5.9	0.01		2	1.7 ± 4.6	0.001	
	T0 OB -T1	6	2.2 ± 6.8	0.47	0 (0%)	14	−1.1 ± 4.9	0.41	2 (14%)

n = number of patients.

### Evaluation of “in-brace results”(T0^OB^-T0^IB^)

Before beginning of treatment ‘in brace’ radiographs showed compared to off brace radiographs a strong reduction of the Cobb angles in kyphosis group for sagittal curves (n = 5, all p < 0.01) and coronal curves (n = 1, p < 0.01). For the scoliosis patients a significant improvement was obtained for 5 sagittal curves and 3 coronal curves. This suggest a maximal reduction of Cobb angles possible at the beginning of treatment.

The right thoracic curve at start with a mean of 43° (range of 14° - 76°) showed an in brace correction with a mean of 28° (range of 14° - 56°), expressed in percentages corresponds this to an achievable reduction of 36% at the beginning of treatment.

For the left lumbar coronal curve the mean Cobb angle was 60° (range 30° - 74°) with a mean in brace correction of 46° (range 22° - 61°), corresponding to a possible reduction of 34%.

For the curves in kyphotic children the constant activation towards extension is reflected in lasting corrections in the sagittal contour in group B.

### Evaluation after one year treatment (T0^OB^-T1)

After one year of brace treatment in kyphosis group the Cobb angles on the radiographs made without brace (T1) compared to beginning of the treatment (T0^OB^) revealed a significant improvement in 3 Cobb angles: 2 sagittal and 1 coronal curve. For the scoliosis group the Cobb angles comparison (T1 -T0^OB^) revealed a significant improvement in 3 sagittal angles for the thoracic, thoraco-lumbar sagittal and pelvic incidence (p < 0.01). Out of this study, the changes in the pelvic incidence can be seen as a secondary event, which follows changes at the primary functional zone of the spine, the thoracolumbar joint.

The progression rate defined as an increase of 6° from baseline (T0^OB^) is shown in Table 2. After one year of brace treatment in one of the 38 patients with scoliosis <25° (group B), a progression was obtained for the thoracolumbar left curve. This demonstrates in essence that in this group of patients the deformity is at least stabilized or even reduced. For the scoliosis patients (group A) the coronal curves showed on average a progression rate of 12.4%, which were higher for the thoracic right and lumbar sagittal curves than for the thoracolumbar left (4.2%) and pelvic obliquity (4.3%). For the sagittal curves the average in progression rate was 1%.

### Questionnaire

**Figure 2:**
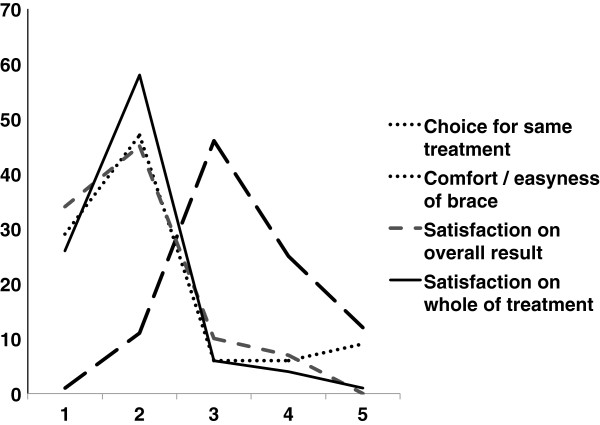
**Results of the questionnaire taken after one year of TLI brace presented graphically.** There was scored for four items related to patient satisfaction. For each item a score had to be chosen from one out of 5 possibilities ranging from 1 to 5. Whole treatment/overall result: 1 = very satisfied; 2 = satisfied; 3= neutral; 4 = unsatisfied; 5 = very unsatisfied; Easiness of wearing: 1 = very good; 2 = good; 3 = fair; 4 = bad; 5 = very bad; Choice for same treatment: 1 = certainly again; 2 = probably again; 3 = Don’t know; 4 = probably not; 5 = certainly not.

Patient information regarding the treatment and satisfaction are shown in Figure 2. After one year TLI treatment most of the patients were satisfied by the visible result, would choose the same treatment again, including postural treatment. One third of the patients perceived a subjective problem with wearing a brace . No child however stopped wearing the brace completely during this first year, so there were no “drop-outs”.

## Discussion

This study evaluated conservative treatment with thoracolumbar lordotic intervention (TLI) in scoliotic and kyphotic deformities in adolescence. In both patient groups after at least one year of treatment a significant improvement was obtained in the sagittal curvature. In addition, the coronal curves showed moderate to good correction, and only rarely progression under TLI treatment. Moreover, the majority of the patients were satisfied with their treatment, and are willing to undergo the same treatment again. This study clearly shows that the initial results of TLI treatment look promising.

It is not excluded that with an earlier start i.e. before the end of the spinal growth one may achieve more with TLI treatment, as the amount of contractures d in joints and real osseous deformations may be less extensive at the start of the treatment. Therefore, starting treatment consequently in an earlier phase of the deforming process, may lead to more success [16,17].

**Figure 3:**
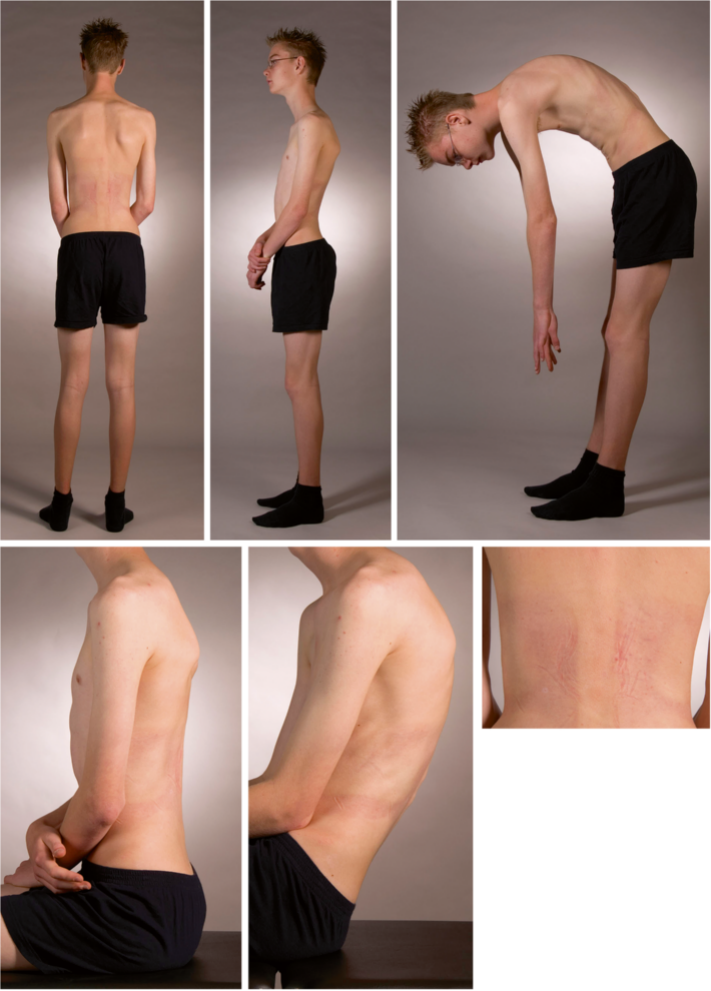
**A fifteen year old boy with progressive Scheuermann kyphosis and serious back pain.** Late referral but first treated with physical therapy between first and second X-ray. Wedging and Schmorl’s nodi mainly in the thoracolumbar area (Cobb Th10-L2: 20°). Picture taken after the first night wearing a TLI brace: Clockwise: Left: standing, some scapular asymmetry is present. Middle: lateral view while active standing upright. Right: the contracted neuromuscular system while bending forward. Note the impossibility to keep the legs vertical, get the lumbar spine horizontal and the visible tight hamstrings. Flexion occurs in the upper spine, not around the hips. Under right : the skin of his back after the first night of wearing a TLI brace. The main pressure is on the paraspinal musculature with the apex at the thoracolumbar area at the same level as the elbow pits. There is no pressure mark on the processi spinosi or the lower ribs. In the middle: sitting with straight legs and left sitting on the edge of the couch. The thoracolumbar kyphosis redresses itself somewhat. TLI will maintain this continuously with progressive reduction.

In the literature, two studies using the original Boston brace technique (conventional TLSO) reveal progression in the sagittal curves [18,19]. Conventional TLSO is characterized by stabilize disease or prevent progression in coronal curves, leading to the conclusion that improvement in the coronal curves is accompanied in derotation in sagittal curves [13]. In the current study improvements of the postural balance were clearly obtained. The changes in the sagittal plane i.e. thoraco-lumbar sagittal, thoracic sagittal, and pelvic incidence angle showed a lasting effect in both patients groups. In a previous study we showed the correction of double major curves while applying a forced lordotic intervention at the thoracolumbar joint [14]. In that study the interdependence of angles in the three dimensional configuration of the spine was demonstrated. The assessment of the sagittal contour and the functional behavior of the spine in any progressing deformity will be helpful (Figure 3).

**Figure 4:**
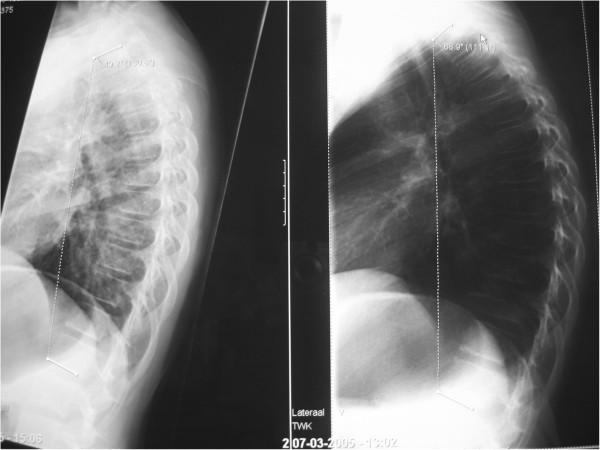
**The effect of sitting on a lateral X-ray.** In these two radiographs of the same girl (12 year, bad posture, fatigue, Scheuermann in elder brother) taken in two different ways of sitting, some confronting chances can be seen. At the left an active way of sitting on the edge of a coach. At the right sitting in a complete relaxed way, showing an increase in the kyphotic curve Th1-Th12 from 49° to 69°. It was known in German literature as a “Sitzbuckel”, but nowadays “gameboyback” looks more appropriate. The anterior compressing forces that will lead via the deformation of soft structures ( Volkmann Hueterprinciple) and deformation of bone ( Wollf’s Law) in a given (genetically) resilience of structures are indirectly illustrated in this manner. Flattening of discs, stiffening of motion segments and wedging of vertebrae will occur if not treated. Loss of muscular condition at the posterior side can lead to further secondary or degenerative changes.

Based on the literature the natural history of the patients in our study with similar age and curve changes may have an expected chance of progression between 40 and 70% [20]. In this context the TLI brace is a major improvement, as progression was a rare event. The failure rate of 21% in this first year in group A included 7 of the 8 referred children with an earlier unsuccessful treatment in a conventional technique.

Technical comparison of TLI brace with other bracing techniques, even with the Milwaukee type of brace, [21,22] shows that in TLI also a type of elongation of the spine is accomplished, but now by a fulcrum, leaving shoulders, cervical spine and head free to move. The intentional correction forces with the Milwaukee brace (at first elongation) and other bracing techniques are essentially directed at the apex of the deformed curves. It will stay difficult to compare results of different types of braces because of many differences in characteristics of technique (Table 3). The TLI treatment puts pressure only in the sagittal plane on the erecting muscles only and acts in the physiologic events of the deformation instead of aiming at the morphological events. It activates by pressure on only the paraspinal muscles these muscles to act more vigorously in achieving a good posture by symmetric extending the spine (Figure 3). Even with the absence of controlateral trochanteric pressure in this brace technique or pure lateral corrective forces on the scoliotic curves, the configuration of the pelvic obliquity changes significantly. So it seems the form and inclination of the pelvis is dependent of the events at the center of the spine like described in evolutionary concepts [23].

**Table 3:** Different types of correcting orthoses for scoliosis and some of the technical characteristics in which they can differ

***Characteristics technique***	**Based on coronal 3 point forces**	**Directed at sagittal contour**	**Symmetric forces**	**Dynamic action required**	**Based on moulds or scans**	**Static /rigid in all Parts of TL spine**	**Meantime adaptations to enhance correction**	**Other indications kyphosis**
CTLSO (Milwaukee)	+	-	-	-	+/−	++	-	+
TLSO (Boston type)	++	+/−	-	-	-	+	-	-
TLSO (Cheneau type)	++	+/−	-	-	+	+	-	-
TLI	-	++	+	+	-	-	++	++
Dynamic (Spine-cor)	-	-	-	++	-	-	+	-
TriaC	+/−	-	-	+	-	-	-	-
Physio-Logic	+	+	+	-	-	-	-	-
Night time overcorrection brace	++	-	-	-	-	+	-	-

These technical differences makes comparison in treatment and results very difficult.

++ = very much; + = much; +/− = depends; - = not.

In the coronal plane a moderate (but not significant) improvement was obtained, suggesting also a lasting effect in the plane perpendicular to the TLI treatment pressure. The adaptation of the TLI brace by the orthotist at control visits towards more lordosis or extension supports the dynamic process of correction with small steps in time during this 12 months treatment period. Actually, the symmetric TLI brace allows movements like lateroflexion, torsion and further extension of the upper part of the body, where only the flexion is prohibited (Figure 4).

The compliance for any brace treatment of spinal deformities in children is crucial. Secondary effects like pain, psychological problems with wearing a brace and the supposed negative feedback by classmates may all influence good compliance [24-26]. Brace treatment should be part of a more complex variety of supportive measurements where subjective scores are important, as children have to live with the brace for a prolonged period of time. Surprisingly, in the current study patient satisfaction was high, with also an apparently acceptable easiness of wearing. In addition, two-third of the patients were willing to undergo the same treatment again. Also the support of the postural therapist was scored as worthwhile. The children received the same message by three health care workers involved in TLI: orthopedic surgeon, orthotist and postural therapist: We all put energy in restoring a good posture with stimulation of extension and prevention of flexion, but you are the decisive factor for success. Thus the improvement of TLI treatment in these children may be explained by the combination of brace plus support.

The concept of thoracolumbar lordotic intervention in our developed brace technique is retrospectively supported by older German, Dutch and Swiss science and corsets compiled in textbooks and since 2001 by the work of Milan Roth of Brno [27-31]. Roth postulated and studied the disproportionate growth relation between the Central Nervous System (“the nervous skeleton”) and bone [32]. He postulated and researched in mechanical models that controlling mechanisms on balance and posture should be arranged around the thoracolumbar joint, where the conus cauda is housed, as this should be as close to the centre of mass as possible. Lordosis at this central point will prevent the spine towards a shortening strategy in oppositional torsional and lateral curvatures. The morphological consequences of Roth hypothesis, that the cord is short and stretched in deformities, was recently confirmed by others with MRI [33-35]. This study may trigger discussion on TLI intervention and contrasts with the current mainstream on accepted aetiology of idiopathic scoliosis. Especially since Dickson describes lordosis in relation to scoliosis to be seen as hazardous, or at least under suspicion. Dickson and followers focus their investigations mainly at the thoracic curves [36]. Recently Ni and colleagues confirmed our discovery of a thoracolumbar kyphosis in scoliotic girls and gave it an important place for future developments in therapeutic strategies [37].

The concept of the corrective mechanism in this study is in line with Wolff’s law on bone remodelling [38,39] and the so called Volkmann-Hueter principle as both are of importance in growth and describe the needed balance in compressive and distractive forces on soft tissues as is guaranteed in playing children and pets [40-42]. That a deviation of the pathway towards optimalisation of growth in otherwise healthy children results in deformations, like kyphosis and scoliosis, must be mainly caused by external, lifestyle depending factors (overloading in flexion)and was written down a century ago in the monoscripts on the causes of “functional” scoliosis and kyphosis by Schanz and Murk Jansen. The latter also presented thorough research on the source of rotational forces in human bodies produced by the asymmetric strength of the diaphragm [43,44]. We think with Roth, that the increased tension in the system (both muscles and nervous tissue)acts as the intrinsic deforming force [45]. Counteract these forces with restoration of lordosis is an attractive alternative for the kyphotic action in many types of braces. Only Weiss advocates a natural lordosis at the lumbar spine in his braces for scoliosis as a prerequisite for true correction and changed the Cheneau type of brace onto a more lordotic configuration at the lumbar spine. But on other etiologic premises and providing forces at a lower area of than in TLI [46,47]. In TLI the important physiologic function of the thoracolumbar joint and its (early) physiologic changes by prolonged sitting with its forward thrust of the upper body and head are leading [48]. In the approach of Weiss promoting natural lordosis at the lumbar spine, the lordotic forces at the lumbar spine and their influence on coronal curves is clear, but is not suitable for correction of, or recommended for kyphotic deformities during childhood. In TLI the sequential adjustments to even more extension in lordosis by adding pads at the thoracolumbar area are not possible in the more static form of rigid braces now in use for different deformities, brought in different designs. Most designs do lock the mobility of the thoracolumbar spine, but can give predictable results, especially in kyphosis.

This study brings new aspects in conservative treatment of adolescent spinal deformities, based on basis biologic and broader clinical knowledge on deformities but has several limitations. First the mean age at start of treatment was quit late due to late time of referral. Some patients were even already treated in another hospital with a conventional Boston brace without success. Part of the explanation of the late diagnosis may be the fast decline of schoolscreening in the Netherlands in the last decades. Secondly we realize, that correction of osseous deformations takes a long time, and a longer follow-up in this group would be helpful to prove the effectiveness of TLI treatment, but this was not possible because of a change of institution by the main author(PvL). Thirdly, although this TLI treatment study was prospective in essence, it was not possible to obtain radiographic images and data from all curves from all patients. Only if all radiological data of the different curves were available or taken in the short period the follow-up study was performed the set of data of the patient was valid and incorporated in this table. Causes of lack of data are i) the absence of X-rays made in other institutions before referral, ii) the wish of parents not to take too much radiographs during the treatment period and iii) cases with technical errors such as incomplete presentation of the spine on the film.

In conclusion, with conservative treatment using thoracolumbar lordotic intervention (TLI) in scoliotic and kyphotic deformities in adolescence the initial results look promising with an effect of corrective force in one plane that effects curves in all other planes. A marked improvement in different planes after one year of treatment is shown. An effect not obtained with current brace techniques.

## Consent

No experimental research is reported. All patients were informed and gave written consent to use their data in a publication at the time they were all interviewed for the questionnaire (MR).

## Competing interests

The authors declare that they have no competing interests.

## Authors’ contributions

PvL developed the technique, treated all patients ,was initiator of the study and drafted the medical parts of the manuscript. MR did as a research student the independent measuring on X-rays , carried out all interviews and gathered all data in files. JK was responsible for the statistical work-out of all data and gave support to the final drafts on results and conclusions. FT was co-iniatiatior and advised in all stadia how to proceed and put the results in a comprehensive article. All authors were involved in reading and approving the final manuscript.

## References

[B1] Lange JE Steen H Brox JI Long-term results after Boston brace treatment in adolescent idiopathic scoliosis Scoliosis 2009 4 17 10.1186/1748-7161-4-17 19709435 PMC2743640

[B2] Olafsson Y Saraste H Soderlund V Hoffsten M Boston brace in the treatment of idiopathic scoliosis J Pediatr Orthop 1995 15 524 527 10.1097/01241398-199507000-00023 7560048

[B3] Peltonen J Poussa M Ylikoski M Three-year results of bracing in scoliosis Acta Orthop Scand 1988 59 487 490 10.3109/17453678809148769 3188850

[B4] Styblo K Conservative treatment of juvenile and adolescent idiopathic scoliosis. A clinical and radiological study on bracing in 290 juvenile and adolescent patients PhD thesis 1991 Nijmegen, Catholic University Nijmegen

[B5] Bradford DS Moe JH Montalvo FJ Winter RB Scheuermann’s Kyphosis and round back deformity. Results of Milwaukee brace treatment J Bone Joint Surg Am 1974 56 740 758 4835819

[B6] Emans JB Kaelin A Bancel P Hall JE Miller ME The Boston bracing system for idiopathic scoliosis. Follow-up results in 295 patients Spine (Phila Pa 1976) 1986 11 792 801 10.1097/00007632-198610000-00009 3810295

[B7] Gutowski WT Renshaw TS Orthotic results in adolescent kyphosis Spine (Phila Pa 1976) 1988 13 485 489 10.1097/00007632-198805000-00009 3187692

[B8] Montgomery SP Erwin WE Scheuermann’s kyphosis--long-term results of Milwaukee braces treatment Spine (Phila Pa 1976) 1981 6 5 8 10.1097/00007632-198101000-00002 6782681

[B9] Pizzutillo PD Nonsurgical treatment of kyphosis Instr Course Lect 2004 53 485 491 15116637

[B10] Weiss HR Turnbull D Bohr S Brace treatment for patients with Scheuermann’s disease - a review of the literature and first experiences with a new brace design Scoliosis 2009 4 22 10.1186/1748-7161-4-22 19788753 PMC2761858

[B11] Rigo M Negrini S Weiss HR Grivas TB Maruyama T Kotwicki T SOSORT consensus paper on brace action: TLSO biomechanics of correction (investigating the rationale for force vector selection) Scoliosis 2006 1 11 10.1186/1748-7161-1-11 16857045 PMC1553475

[B12] Negrini S Zaina F Atanasio S BRACE MAP, a proposal for a new classification of braces Stud Health Technol Inform 2008 140 299 302 18810040

[B13] Negrini S Minozzi S Bettany-Saltikov J Zaina F Chockalingam N Grivas TB Kotwicki T Maruyama T Romano M Vasiliadis ES Braces for idiopathic scoliosis in adolescents Spine (Phila Pa 1976) 2010 35 1285 1293 20461027 10.1097/BRS.0b013e3181dc48f4

[B14] van Loon PJM Kühbauch BAG Thunnissen FB Forced lordosis on the thoracolumbar junction can correct coronal plane deformity in adolescents with double major curve pattern idiopathic scoliosis Spine (Phila Pa 1976) 2008 33 797 801 10.1097/BRS.0b013e3181694ff5 18379408

[B15] Richards BS Bernstein RM D’Amato CR Thompson GH Standardization of criteria for adolescent idiopathic scoliosis brace studies: SRS committee on bracing and nonoperative management Spine (Phila Pa 1976) 2005 30 2068 2075 10.1097/01.brs.0000178819.90239.d0 16166897

[B16] Mehta MH The conservative management of juvenile idiopathic scoliosis Acta Orthop Belg 1992 58 Suppl 1 91 97 1456025

[B17] Stokes IA Krag MH Wilder DG Critique of the optimum spine Spine (Phila Pa 1976) 1988 13 597 10.1097/00007632-198805000-00030 3187708

[B18] Labelle H Dansereau J Bellefleur C Poitras B Three-dimensional effect of the Boston brace on the thoracic spine and rib cage Spine (Phila Pa 1976) 1996 21 59 64 10.1097/00007632-199601010-00013 9122764

[B19] Perie D Aubin CE Petit Y Beausejour M Dansereau J Labelle H Boston brace correction in idiopathic scoliosis: a biomechanical study Spine (Phila Pa 1976) 2003 28 1672 1677 12897490 10.1097/01.BRS.0000083165.93936.6D

[B20] Peterson LE Nachemson AL Prediction of progression of the curve in girls who have adolescent idiopathic scoliosis of moderate severity. Logistic regression analysis based on data from the brace study of the scoliosis research society J Bone Joint Surg Am 1995 77 823 827 7782354 10.2106/00004623-199506000-00002

[B21] Andrews G MacEwen GD Idiopathic scoliosis. An 11-year follow-up study of the role of the Milwaukee brace in curve control and trunco-pelvic alignment Orthopedics 1989 12 809 816 2740262 10.3928/0147-7447-19890601-06

[B22] Howard A Wright JG Hedden D A comparative study of TLSO, Charleston, and Milwaukee braces for idiopathic scoliosis Spine (Phila Pa 1976) 1998 23 2404 2411 10.1097/00007632-199811150-00009 9836354

[B23] Gracovetsky S Farfan H The optimum spine Spine (Phila Pa 1976) 1986 11 543 573 10.1097/00007632-198607000-00006 3538436

[B24] Mak I Lou E Raso JV Hill DL Parent E Mahood JK Moreau MJ Hedden D The effect of time on qualitative compliance in brace treatment for AIS Prosthet Orthot Int 2008 32 136 144 10.1080/03093640802015839 18569881

[B25] Morton A Riddle R Buchanan R Katz D Birch J Accuracy in the prediction and estimation of adherence to bracewear before and during treatment of adolescent idiopathic scoliosis J Pediatr Orthop 2008 28 336 341 10.1097/BPO.0b013e318168d154 18362800

[B26] Vandal S Rivard CH Bradet R Measuring the compliance behavior of adolescents wearing orthopedic braces Issues Compr Pediatr Nurs 1999 22 59 73 10.1080/014608699265293 10786513

[B27] Roth M Neurovertebral and osteoneural growth relations. A concept of normal and pathological development of the skeleton 1985 First Brno, Czech Republique

[B28] Roth M Idiopathic scoliosis caused by a short spinal cord Acta Radiol Diagn (Stockh) 1968 7 257 271 4883824 10.1177/028418516800700308

[B29] Roth M Models of vertebro-neural relations Acta Radiol Diagn (Stockh) 1969 9 746 753 5381077

[B30] Roth M Idiopathic scoliosis and Scheuermann’s disease: essentially identical manifestations of neuro-vertebral growth disproportion Radiol Diagn (Berl) 1981 22 380 391 7330209

[B31] Hohmann G Hackenbroch M Lindemann K Handbuch der Orthopaedie (6vol) 1956 Georg Thieme Verlag, Stuttgart vol I and II

[B32] van Loon PJM van Rhijn LW The central cord-nervous roots complex and the formation and deformation of the spine; the scientific work on systematic body growth by Milan Roth of Brno (1926–2006) Stud Health Technol Inform 2008 140 170 186 18810022

[B33] Chu WC Lam WM Ng BK Tze-Ping L Lee KM Guo X Cheng JC Burwell RG Dangerfield PH Jaspan T Relative shortening and functional tethering of spinal cord in adolescent scoliosis - Result of asynchronous neuro-osseous growth, summary of an electronic focus group debate of the IBSE Scoliosis 2008 3 8 10.1186/1748-7161-3-8 18588673 PMC2474583

[B34] Chu WCW Man GCW Lam WWM Yeung BHY Chau WW Ng BKW Lam T Lee KM Cheng JCY Morphological and functional electrophysiological evidence of relative spinal cord tethering in adolescent idiopathic scoliosis Spine (Phila Pa 1976) 2008 33 673 680 10.1097/BRS.0b013e318166aa58 18344862

[B35] Chu WC Ng BK Li AM Lam T Lam WW Cheng JC Dynamic magnetic resonance imaging in assessing lung function in adolescent idiopathic scoliosis: a pilot study of comparison before and after posterior spinal fusion J Orthop Surg Res 2007 2 20 10.1186/1749-799X-2-20 18021435 PMC2203977

[B36] Dickson RA The aetiology of spinal deformities Lancet 1988 1 1151 1155 2896969 10.1016/s0140-6736(88)91963-0

[B37] Ni H Zhu X He S Yang C Wang C Zhao Y Wu D Xu J Li M An increased kyphosis of the thoracolumbar junction is correlated to more axial vertebral rotation in thoracolumbar/lumbar adolescent idiopathic scoliosis Spine (Phila Pa 1976) 2010 35 E1334 E1338 20736888 10.1097/BRS.0b013e3181e5370b

[B38] Hert J Wolff’s law of transformation after 100 years Acta Chir Orthop Traumatol Cech 1990 57 465 476 2091427

[B39] Pearson OM Lieberman DE The aging of Wolff’s “law”: ontogeny and responses to mechanical loading in cortical bone Am J Phys Anthropol 2004 39 Suppl 63 99 15605390 10.1002/ajpa.20155

[B40] Castro FPJ Adolescent idiopathic scoliosis, bracing, and the Hueter-Volkmann principle Spine J 2003 3 180 185 10.1016/S1529-9430(02)00557-0 14589197

[B41] Grivas TB Vasiliadis ES Rodopoulos G Bardakos N The role of the intervertebral disc in correction of scoliotic curves. A theoretical model of idiopathic scoliosis pathogenesis Stud Health Technol Inform 2008 140 33 36 18809995

[B42] Stokes IAF Mechanical effects on skeletal growth J Musculoskelet Neuronal Interact 2002 2 277 280 15758453

[B43] Schanz A The static load-dependant deformities of the spine, especially the scoliosis in children (inGerman) 1904 Ferdinand Enke Verlag, Stuttgart

[B44] Jansen M The physiologic scoliosis and its cause (in Dutch) 1912 E.J.Brill, publisher, Leiden

[B45] van Loon PJM Clinical detectable tension in the growing body Stud Health Technol Inform 2008 140 52 18809999

[B46] Weiss HR Ein neues Korsett zur Behandlung der Idiopathischen Skoliose und anderer Wirbelsäulendeformitaten Orthopädie-Technik 2004 55 808 814

[B47] Weiss HR Dallmayer R Gallo D Sagittal counter forces (SCF) in the treatment of idiopathic scoliosis; a preliminary report Pediatr Rehabil 2006 9 24 30 16352502 10.1080/13638490500038126

[B48] van Loon PJM Grivas TB Scoliosis idiopathic? The etiologic factors in scoliosis will affect preventive and conservative therapeutic strategies Recent advances in scoliosis 2012 1 InTech, Rijeka 211 234

